# *Veillonella* and *Streptococcus* are associated with aging of the gut microbiota and affect the efficacy of immune checkpoint inhibitors

**DOI:** 10.3389/fimmu.2025.1528521

**Published:** 2025-05-22

**Authors:** Yuya Hirasawa, Junya Isobe, Masahiro Hosonuma, Toshiaki Tsurui, Yuta Baba, Eiji Funayama, Kohei Tajima, Masakazu Murayama, Yoichiro Narikawa, Hitoshi Toyoda, Midori Shida, Aya Sasaki, Yuuki Maruyama, Yasunobu Amari, Emiko Mura, Risako Suzuki, Nana Iriguchi, Tomoyuki Ishiguro, Ryotaro Ohkuma, Masahiro Shimokawa, Hirotsugu Ariizumi, Yutaro Kubota, Atsushi Horiike, Takehiko Sambe, Naoki Uchida, Satoshi Wada, Shinichi Kobayashi, Yuji Kiuchi, Atsuo Kuramasu, Kiyoshi Yoshimura, Takuya Tsunoda

**Affiliations:** ^1^ Division of Medical Oncology, Department of Medicine, Showa Medical University School of Medicine, Tokyo, Japan; ^2^ Department of Hospital Pharmaceutics, School of Pharmacy, Showa Medical University, Tokyo, Japan; ^3^ Department of Clinical Immuno-Oncology, Clinical Research Institute for Clinical Pharmacology and Therapeutics, Showa Medical University, Tokyo, Japan; ^4^ Department of Pharmacology, Showa Medical University Graduate School of Medicine, Tokyo, Japan; ^5^ Pharmacological Research Center, Showa Medical University, Tokyo, Japan; ^6^ Department of Hematology, Showa Medical University Fujigaoka Hospital, Kanagawa, Japan; ^7^ Department of Pharmacology, Showa Medical University School of Pharmacy, Tokyo, Japan; ^8^ Department of Gastroenterological Surgery, Tokai University School of Medicine, Kanagawa, Japan; ^9^ Department of Otorhinolaryngology-Head and Neck Surgery, Showa Medical University School of Medicine, Tokyo, Japan; ^10^ Department of Otorhinolaryngology, Showa Medical University Fujigaoka Hospital, Kanagawa, Japan; ^11^ Department of Orthopedic Surgery, School of Medicine, Showa Medical University, Tokyo, Japan; ^12^ Division of Clinical Pharmacology, Department of Pharmacology, Showa Medical University School of Medicine, Tokyo, Japan; ^13^ Clinical Research Institute for Clinical Pharmacology and Therapeutics, Showa Medical University, Tokyo, Japan; ^14^ Department of Clinical Diagnostic Oncology, Clinical Research Institute for Clinical Pharmacology and Therapeutics, Showa Medical University, Tokyo, Japan

**Keywords:** gut microbiota, immune checkpoint inhibitor, aging, *Veillonella*, *Streptococcus*

## Abstract

**Introduction:**

The rapid increase in the number of elderly patients with cancer necessitates treatment strategies based on the effects of aging because of drastic side effects of cytotoxic anticancer agents. Immune checkpoint inhibitors (ICIs) are relatively less toxic and can be easily administered to vulnerable and aged patients suffering from cancer. The diversity of gut microbiota and specific bacteria affects the efficacy and safety of ICIs. Therefore, this study aimed to assess the effect of aging on gut microbiota that play crucial roles in determining antitumor efficacy of drugs.

**Methods:**

Stool samples were collected from 36 aged patients pathologically diagnosed with solid tumors before the start of drug therapy, and gut microbial composition was analyzed using next generation sequencing. The association between gut microbiota and efficacy and safety of ICIs was analyzed.

**Results:**

The abundance of *Veillonella* species significantly decreased in patients aged ≥75 years. Additionally, the gut microbiota in the responder group was significantly higher than that in the non-responder group regardless of age. The abundance of *Streptococcus* species was significantly higher in the responder group than that in the non-responder group.

**Conclusions:**

These gut microbiota changes with aging, and its characteristics are important parameters that also affect the efficacy of ICIs.

## Introduction

The global population is aging, and the number of elderly patients with cancer is increasing. Elderly patients have unique characteristics, such as decreased function of major organs owing to aging, increased susceptibility to adverse events, multiple comorbidities, increased number of medications, and cognitive decline (1). Elderly patients with cancer are often excluded from clinical trials, resulting in a lack of evidence to prescribe these medications to them (2, 3). Therefore, elderly patients with cancer are intolerant to pharmacotherapy, particularly cytotoxic agents. However, treatment should not be withheld based on calendar age alone, and the performance status (PS) and Comprehensive Geriatric Assessment (CGA), which includes physical and cognitive functions as represented by the G8, should be considered (4, 5). However, PS and CGA depend on subjective elements, and no simple and objective evaluation index for the effects of aging has yet been established. Therefore, clinical trials and research on the treatment of elderly patients with cancer are necessary (6). With the introduction of immune checkpoint inhibitors (ICIs) in 2014, drugs that use the patient’s own immune system have become key elements for pharmacotherapy. ICIs are less toxic than conventional cell-killing anticancer drugs, and may be administered to elderly populations. Age-related immune aging and changes in the composition of gut microbiota may affect antitumor immunity; however, the actual situation is not yet clear.

Immune function, particularly acquired immune response, declines with aging, which is known as immunosenescence (7–9). In immunosenescence, T cells are severely affected (10–13). As T cells age, aging of naïve T cells reduces responsiveness to neoantigens, which may adversely affect the efficacy of ICIs. In some studies, no survival benefit with ICIs has been observed in patients aged ≥75 years (14, 15), and a pooled analysis of clinical trials using pembrolizumab in non-small cell lung cancer has indicated a significantly high incidence of grade 3 or higher immune-related adverse events (irAEs) in patients aged ≥75 years (16). Clinically, these results suggest that immune aging affects antitumor immunity in patients aged ≥75 years.

Approximately 40 trillion gut microbes are present in our intestinal tract (17, 18). Gut bacteria are involved in physiological homeostasis, progression and treatment of various diseases, such as allergic diseases and inflammatory bowel disease, and cancer development and therapeutic efficacy (19–21). In malignant melanoma, patients consuming >20 g fiber per day are more likely to have better efficacy of ICIs via modification of gut bacteria (22). For example, in malignant melanoma and lung cancer, specific gut microbiota have been shown to be predictive factors for ICIs (23, 24). *Akkermansia* and *Bifidobacterium* can modulate immune response to cancer and enhance the therapeutic efficacy of ICIs (23, 25). *Turicibacter* and *Acidaminococcus* affect the efficacy of ICIs as observed in a Japanese cohort (26, 27).

The gut microbiota remains stable during adulthood and then changes in old age because of age-related physiological decline and dietary changes (28). The gut microbiota of the elderly show higher individual variation, diversity, and decreased stability than does those of the adults (29). Gut microbial composition of the elderly shows an increase in bacterial population belonging to the phylum Bacteroidetes and a decrease in *Bifidobacterium* and *Bacteroides*, which produce short-chain fatty acids (30–32).Moreover, the gut microbiota of healthy subjects aged between 0–104 years change to an elderly type around the age of 70 years, with an increase in the abundance of Proteobacteria phylum and a decrease in Firmicutes phylum (33).

Although the gut microbiota differs between young and elderly people, age-related changes in the gut microbiota of patients with cancer are unknown. Diversity of the gut microbiota and specific bacterial population affect the efficacy and safety of ICIs in patients with cancer; however, the effects of age-related changes in gut microbiota on antitumor immunity are largely unexplored.

In this study, we analyzed the gut microbiota of patients with cancer by age group to analyze age-related changes in the gut microbiota and their effects on the efficacy and side effects of ICIs.

## Materials and methods

### Patients

Thirty-six patients with cancer aged 50 to 83 years, who were pathologically diagnosed with solid tumors and have undergone systemic drug therapy from February 2019 to January 2021 were included in the study. Stool samples were collected before the start of drug therapy. The patients were divided among age groups between 50–59 (50s; n, 10), 60–69 (60s; n, 7), 70–79 (70s; n, 18), and 80–89 (80s; n, 1). The cancer types included stomach cancer (15 patients), non-small cell lung cancer (six patients), colorectal cancer (six patients), microsatellite instability-high (MSI-H) solid cancer (three patients), esophageal cancer (three patients), renal cancer (two patients), and pancreatic cancer (one patient) (**Table 1**).

**Table 1:** Baseline characteristic of the patients (n=36).

Patient characteristics	Number of cases (%)
Cancer Type	
Gastric cancer	15 (41.6)
Non-small cell lung cancer	6 (16.7)
Colorectal cancer	6 (16.7)
Esophageal cancer	3 (8.3)
MSI-H cancer	3 (8.3)
Renal cell carcinoma	2 (5.6)
Pancreatic cancer	1 (2.8)
Age groups (years)	
50–59	10 (27.8)
60–69	7 (19.4)
70–79	18 (50.0)
80–89	1 (2.8)

MSI-H, microsatellite instability-high.

Of these, 16 patients were treated with ICI alone. ICI treatment consisted of nivolumab alone in 11 patients, pembrolizumab alone in three patients, and a combination of nivolumab and ipilimumab in two patients. Five patients were in their 50s, three in their 60s, seven in their 70s, and one in his 80s. **Table 2** shows baseline characteristics and clinical outcomes of their efficacy and safety.

**Table 2:** Characteristic of the patients treated with ICIs (n=16).

Patient characteristics	Number of cases (%)
Cancer Type	
Gastric cancer	7 (43.6)
MSI-H cancer	3 (18.8)
Non-small cell lung cancer	2 (12.5)
Esophageal cancer	2 (12.5)
Renal cell carcinoma	2 (12.5)
Regimen	
Nivolumab	11 (68.8)
Pembrolizumab	3 (18.8)
Nivolumab + Ipilimumab	2 (12.5)
Age groups (years)	
50–59	5 (31.2)
60–69	3 (18.8)
70–79	7 (43.6)
80–89	1 (6.3)
Progression-free survival	
< 120 days	8 (50.0)
≥ 120 days	8 (50.0)
Disease control rate	
SD-CR (non-PD group)	9 (56.2)
PD (PD group)	7 (43.8)
irAEs	
Non-incident	7 (43.8)
Incident	9 (56.2)

MSI-H, microsatellite instability-high; SD, stable disease; CR, complete response; PD, progressive disease; irAEs, immune-related adverse events.

Gut microbiota was analyzed by next generation sequencing, and those of different age groups were compared. For the 16 patients treated with ICI alone, the association between gut microbiota and efficacy/safety was analyzed. This study was approved by the Ethics Committee of Showa University School of Medicine (approval No. B-2018-022). All patients provided written informed consent for their participation in this study.

### DNA extraction from feces

Feces samples were collected from each patient using a stool collection kit containing guanidine (TechnoSuruga Laboratory, Shizuoka, Japan) and stored at -80 °C until analysis. DNA was extracted from those samples using a QIAamp PowerFecal Pro DNA Kit (Qiagen, Hilden, Germany) according to the manufacturer’s instructions.

### Metagenome analysis

Metagenome analysis was performed using a next-generation sequencer (MySeq; Illumina, San Diego, CA, USA) to analyze the 16S V3 and V4 regions of ribosomal RNA genes. Sequencing data in FASTQ format were imported into QIIME2 v.2021.4 (https://docs.qiime2.org/2021.4/), quality-controlled with QIIME2 DADA2 plug-in, and explored for downstream analysis using FeatureTable artifact. A rooted phylogenetic tree for alpha diversity analysis was generated using q2-phylogeny plugin. Reads from each sample were rarefied to a depth of 5,000–10,000 to minimize the effect of sequencing depth on alpha and beta diversity measures. Based on the obtained 16S rRNA sequence data, taxonomic and compositional analyses were performed using the plugins q2-feature-classifier, q2-taxa, and the R package QIIME2R (https://github.com/jbisanz/qiime2R).

### Statistical analysis

We analyzed genus-level differences in gut microbiota between two groups defined by age or clinical outcomes. Differential abundance analyses were performed using relative abundance data generated with QIIME2, and statistical significance between groups was evaluated using the Mann–Whitney U test in R v.4.0.5 (https://www.r-project.org/) running under RStudio v.1.4.1106 (https://download1.rstudio.org/desktop/windows/RStudio-1.4.1106.exe). P-values less than 0.05 were considered statistically significant. QIIME2 was used to generate α-diversity and β-diversity indices, as well as genus-level relative abundance data for differential abundance testing. PICRUSt2 was used to generate a MetaCyc metabolic pathway to predict the functions of 16S rRNA sequences in each sample. For functional profiling, predicted MetaCyc pathways generated by PICRUSt2 were also compared between groups using the Mann–Whitney U-test. A volcano plot was generated to visualize pathways with significant differences in predicted functional activity (p < 0.05, |log2 fold change| > 1).

In the analysis of the association between gut microbiota and efficacy/safety for the 16 patients treated with ICI alone, efficacy was evaluated based on progression-free survival (PFS) and best overall response (BOR). The PFS was classified into 2 groups with a cutoff of 120 days: PFS ≥ 120 days group and PFS < 120 days group. The BOR was classified into 2 groups based on disease control rate (DCR): non-PD group (SD-CR) and PD group. Safety was evaluated based on irAEs, and classified into 2 groups according to presence/absence of irAEs: incident group and non-incident group.

## Results

### Gut microbiota of patients grouped by the age of 75

**Figure 1:**
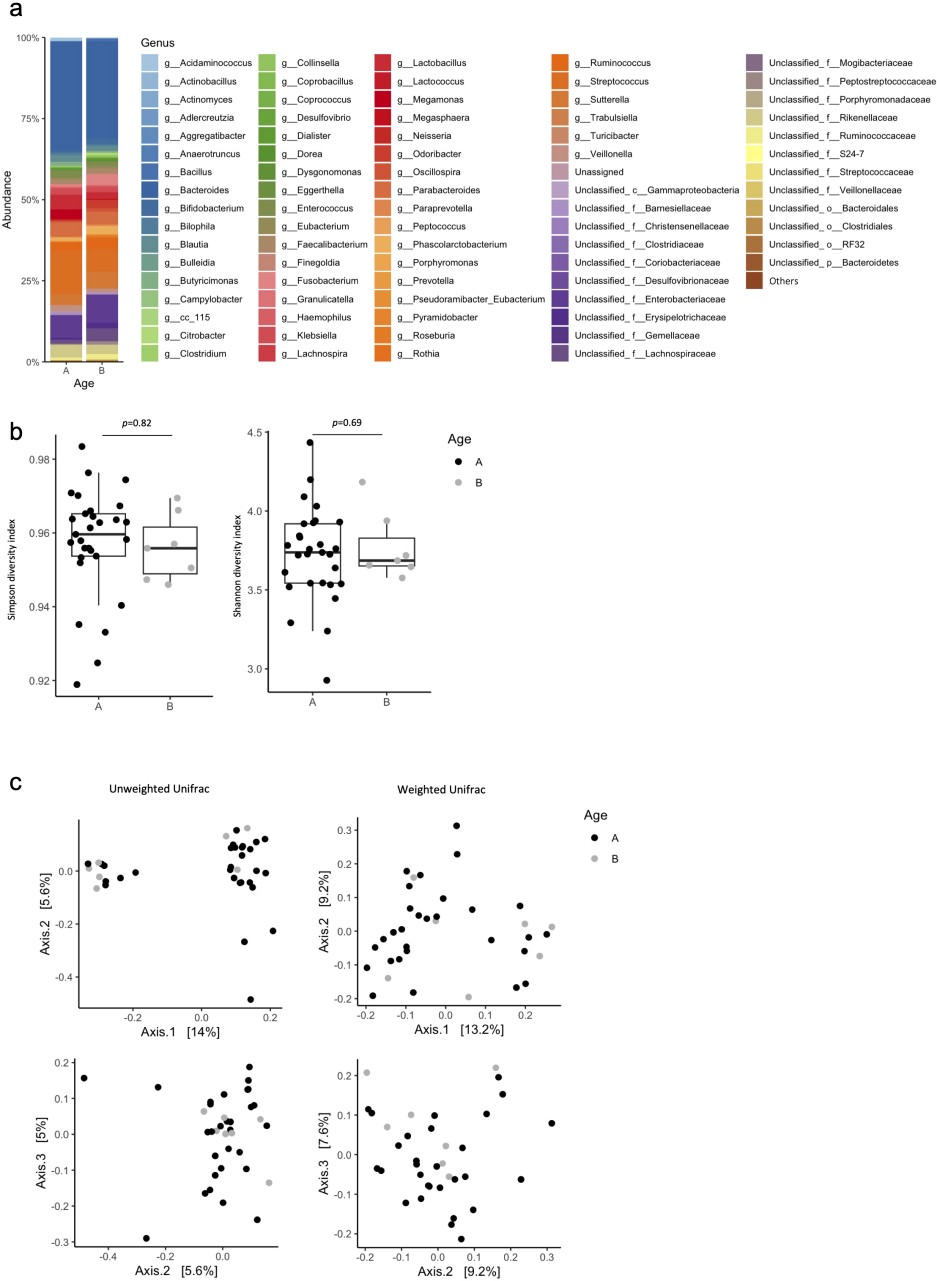
Percentage composition of microbiota in the Age <75 and Age ≥75 groups. **(a)** Relative abundance of bacteria at the genus level in the Age <75 **(A)** and Age ≥75 **(B)** groups. Bacteria found in more than 0.1% cases were summed up to 100%. **(b)** Box plot of Simpson’s index and Shannon index in the Age <75 **(A)** and Age≥75 **(B)** groups. **(c)** Principal coordinate analysis (PCoA) based on the Bray–Curtis dissimilarity displaying variations in bacterial abundance in the Age <75 **(A)** and Age ≥75 **(B)** groups. Statistical differences were calculated by analyzing similarity algorithm.

The gut microbiota at the genus level in patients aged <75 years (Age<75) and ≥75 years (Age≥75) groups were plotted (**Figure 1a**). The results of the α-diversity and β-diversity were used to compare the diversity of each flora, and no differences in diversity were observed between groups (**Figures 1b, c**).

### Bacterial abundance in the flora grouped by age 75

**Figure 2:**
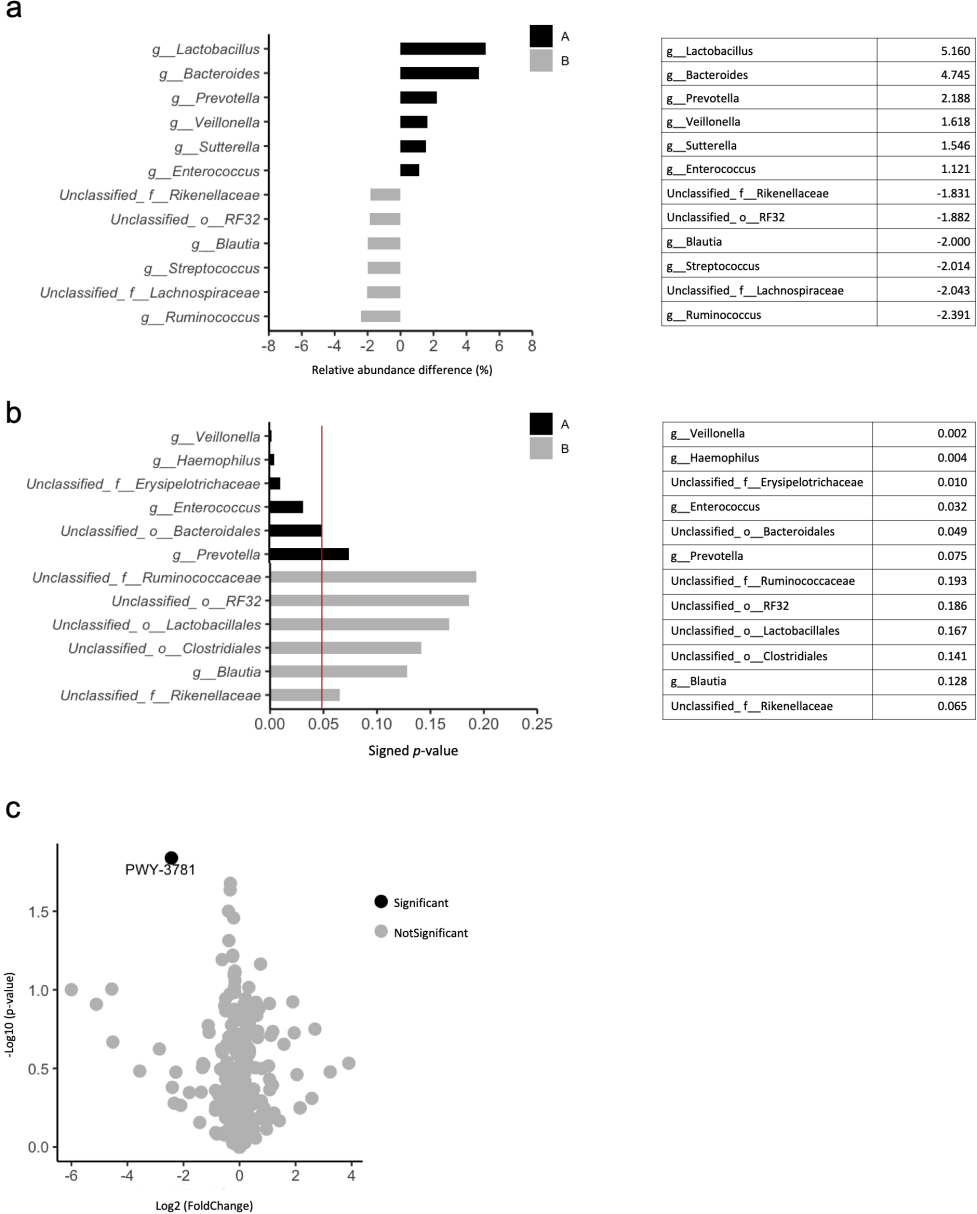
Differences in composition of gut microbiota in the Age <75 and Age ≥75 groups. **(a)** Relative abundance of bacteria in the Age <75 **(A)** and Age ≥75 **(B)** groups. The six top bacteria at the genus level. **(b)** Statistical significance of differences in bacterial abundance between the Age <75 **(A)** and Age ≥75 **(B)** groups using the Mann–Whitney U test. The red line indicates a p-value of 0.05. The six top bacteria at the genus level. **(c)** Volcano plots showing bacterial metabolic pathways that differ between the Age <75 and Age ≥75 groups.

Analysis of bacterial abundances at the genus level for gut microbiota showed that in the Age<75 group, *Lactobacillus* (5.160%), *Bacteroides* (4.745%), *Prevotella* (2.188%), *Veillonella* (1.618%), *Sutterella* (1.546%), and *Enterococcus* (1.121%) were frequently detected (**Figure 2a**). In the Age≥75 group, *Ruminococcus* (2.391%), *Streptococcus* (2.014%), and *Blautia* (2.000%) were frequently detected (**Figure 2a**). In the Age<75 group, *Veillonella* (*p*=0.002) and *Haemophilus* (*p*=0.004) were detected more frequently than in the Age≥75 group (**Figure 2**). Only *Veillonella* was detected more abundantly in the Age<75 group than in the Age≥75 group.

Functional analysis of genes showed that *PWY-3781* associated with aerobic respiration was more common in the flora of Age≥75 group than that in the Age<75 group (*p*=0.01) (**Figure 2c**).

### Gut microbiota grouped by DCR, PFS, and irAE

**Figure 3:**
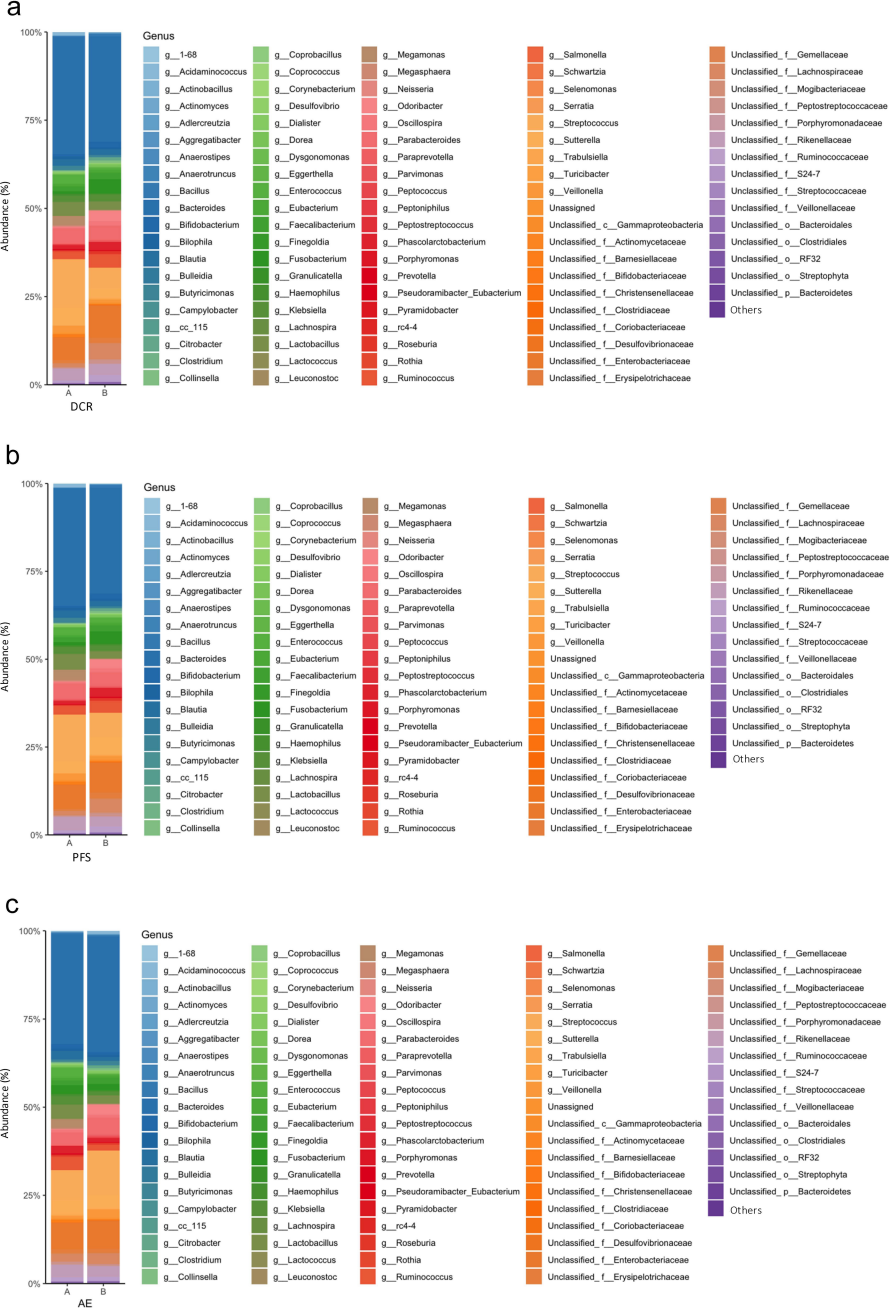
Percentage composition of microbiota in the treatment effects and adverse events groups. **(a)** Relative abundance of bacteria at the genus level in the non-PD **(A)** and PD **(B)** groups. Bacteria found in more than 0.1% cases were summed up to 100%. **(b)** Relative abundance of bacteria at the genus level in the PFS ≥120 days **(A)** and PFS <120 days **(B)** groups. Bacteria found in more than 0.1% cases were summed up to 100%. **(c)** Relative abundance of bacteria at the genus level in the incident **(A)** and non-incident **(B)** groups. Bacteria found in more than 0.1% cases were summed up to 100%. PD, Progressive Disease; PFS, progression-free survival.

Gut microbiota at the genus level in the non-PD and PD groups for DCR, in the ≥120 days and <120 days groups for PFS, and in the groups with and without irAE are shown in the bar plot (**Figures 3a–c**).

**Figure 4:**
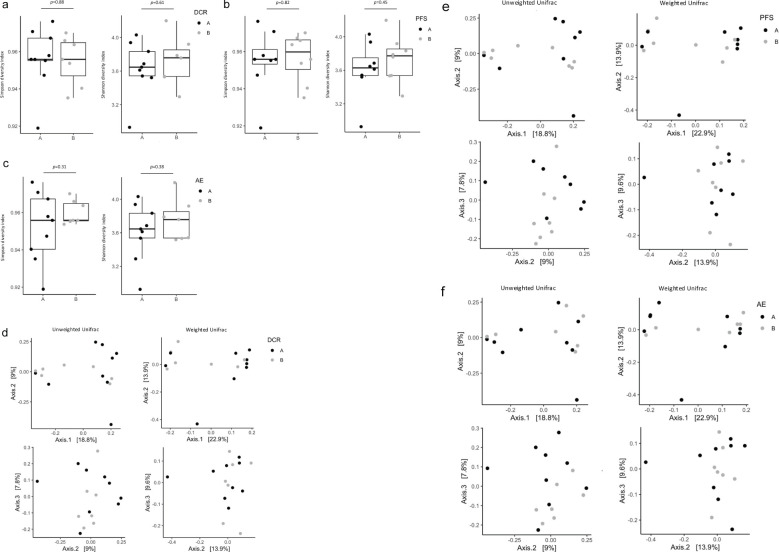
Diversity of microbiota in treatment effects and adverse events groups. **(a)** Box plot of Simpson’s index and Shannon index in the non-PD **(A)** and PD **(B)** groups. **(b)** Box plot of Simpson’s index and Shannon index in the PFS ≥120 days **(A)** and PFS <120 days **(B)** groups. **(c)** Box plot of Simpson’s index and Shannon index in the incident **(A)** and non-incident **(B)** groups. **(d)** PCoA based on the Bray–Curtis dissimilarity displaying bacterial variations in the non-PD **(A)** and PD **(B)** groups. Statistical differences were calculated by analyzing similarity algorithm. **(e)** PCoA based on the Bray–Curtis dissimilarity displaying bacterial variations in the PFS ≥120 days **(A)** and PFS <120 days **(B)** groups. Statistical differences were calculated by analyzing similarity algorithm. **(f)** PCoA based on the Bray–Curtis dissimilarity displaying bacterial variations in the incident **(A)** and non-incident **(B)** groups. Statistical differences were calculated by analyzing similarity algorithm. PD, progressive disease; PFS, progression-free survival; PCoA, principal coordinate analysis.

To compare the diversity of each flora, α-diversity and β-diversity were assessed. The results showed no differences in diversity between the two groups of each classification, including DCR, PFS, and irAE. (**Figures 4a–f**).

**Figure 5:**
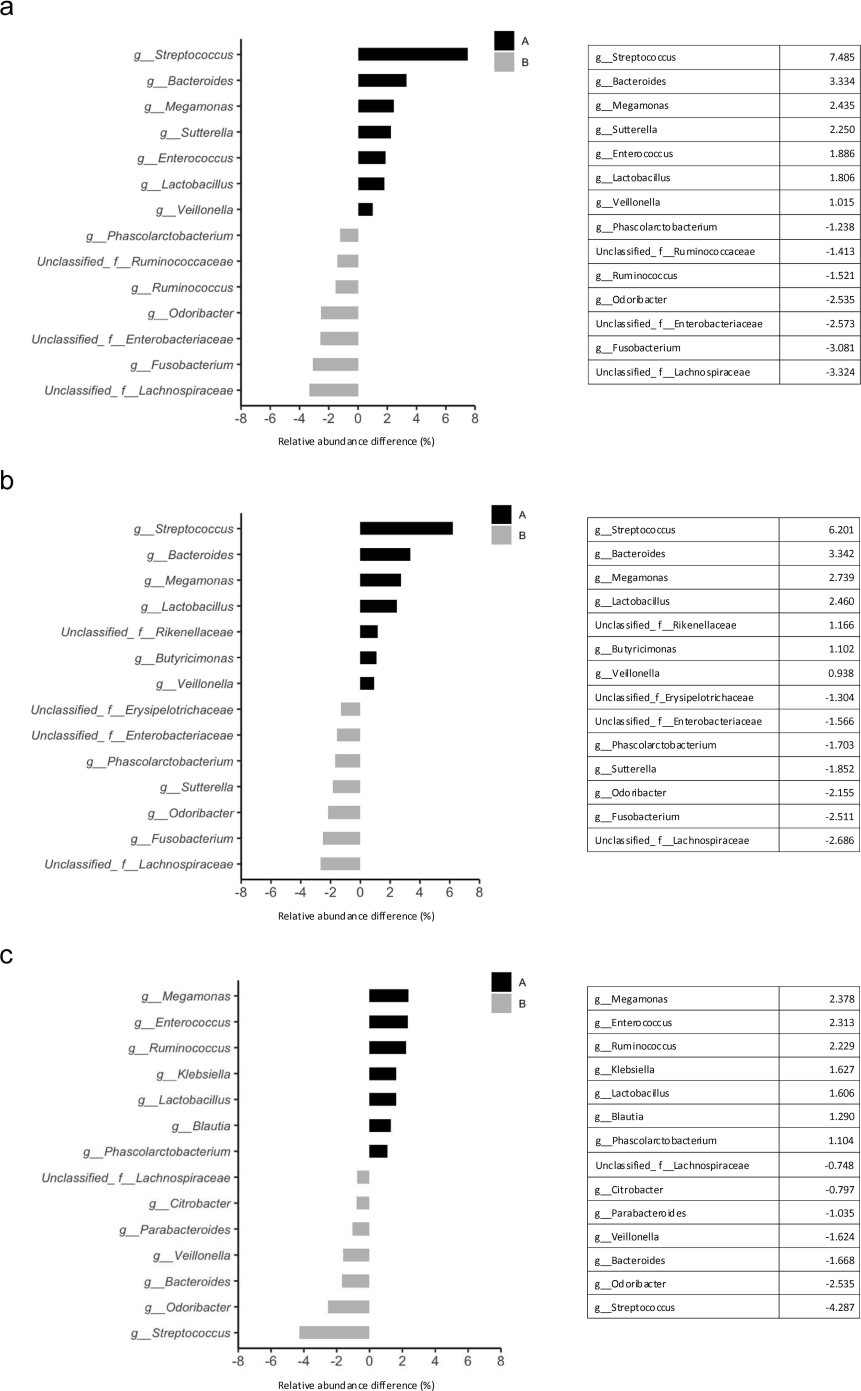
Differences in gut microbial compositions between the treatment effects and adverse events groups. **(a)** Relative abundance of bacteria between the non-PD **(A)** and PD **(B)** groups. The seven top bacteria at the genus level. **(b)** Relative abundance of bacteria in the PFS ≥120 days **(A)** and PFS <120 days **(B)** groups. The seven top bacteria at the genus level. **(c)** Relative abundance of bacteria in the incident **(A)** and non-incident **(B)** groups. The seven top bacteria at the genus level. PD, progressive disease; PFS, progression-free survival.

Genus-level analysis of the differences in gut microbiota between the non-PD and PD groups showed that the non-PD group frequently contained *Streptococcus* (7.485%), *Bacteroides* (3.334%), *Megamonas* (2.435%), *Sutterella* (2.250%), *Enterococcus* (1.886%), *Lactobacillus* (1.806%), and *Veillonella* (1.015%) (**Figure 5a**). *Fusobacterium* (3.081%), *Odoribacter* (2.535%), *Ruminococcus* (1.521%), and *Phascolarctobacterium* (1.238%) were frequently detected in the PD group (**Figure 5a**).

Genus-level analysis of the differences in gut microbiota between the groups with PFS ≥120 days and PFS <120 days showed that *Streptococcus* (6.201%), *Bacteroides* (3.342%), *Megamonas* (2.739%), *Lactobacillus* (2.460%), *Butyricimonas* (1.102%), and *Veillonella* (0.938%) were frequently detected in the ≥120 days group (**Figure 5b**). *Fusobacterium* (2.511%), *Odoribacter* (2.155%), *Sutterella* (1.852%), and *Phascolarctobacterium* (1.703%) were frequently detected in the <120 days group (**Figure 5b**).

Genus-level analysis of the differences in gut microbiota between the groups with and without irAE showed that *Megamonas* (2.378%), *Enterococcus* (2.313%), *Ruminococcus* (2.229%), *Klebsiella* (1.627%), *Lactobacillus* (1.606%), *Blautia* (1.290%), and *Phascolarctobacterium* (1.104%) were frequently detected in the incident group (**Figure 5c**). *Streptococcus* (4.287%), *Odoribacter* (2.535%), *Bacteroides* (1.668%), *Veillonella* (1.624%), *Parabacteroides* (1.035%), and *Citrobacter* (0.797%) were frequently detected in the non-incident group (**Figure 5c**).

**Figure 6:**
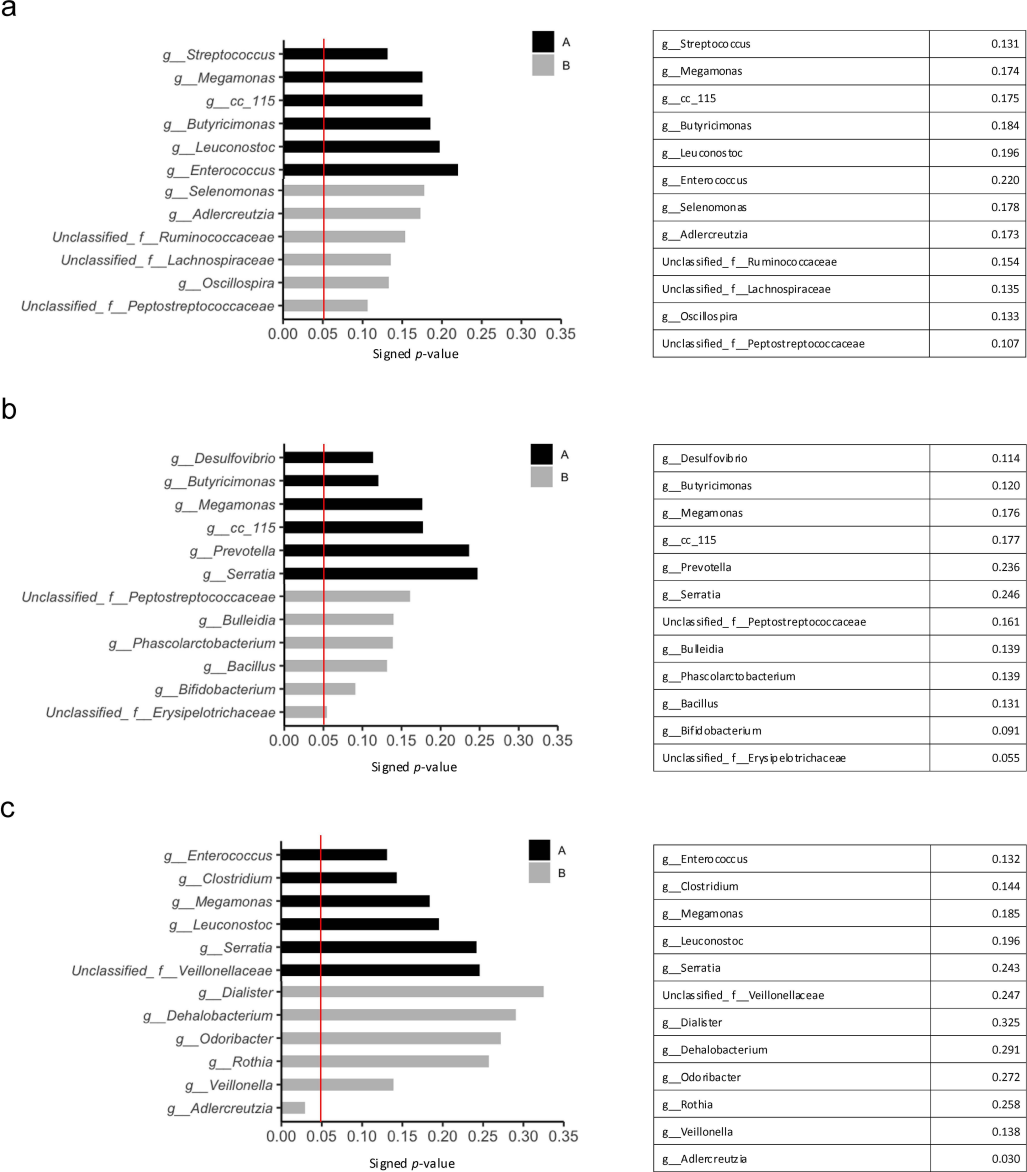
Statistically significant differences in the treatment effects and adverse events groups using the Mann–Whitney U test. The red line indicates a p-value of 0.05. **(a)** The six top bacteria in the non-PD **(A)** and PD **(B)** groups at the genus level. **(b)** The six top bacteria in the PFS ≥120 days **(A)** and PFS <120 days **(B)** groups at genus level. **(c)** The six top bacteria in the incident **(A)** and non-incident **(B)** groups at the genus level. PD, progressive disease; PFS, progression-free survival.

Based on the results of the Mann–Whitney U test, only *Adlercreutzia* was significantly more abundant in the non-incident group of irAE compared to the incident group (p=0.03) (**Figures 6a–c**).

## Discussion

Given the increasing number of reports on the efficacy and safety analysis of clinical ICIs using age 75 as a cutoff, we analyzed based on the hypothesis that differences in gut microbiota would be observed between the age groups ≥75 years and < 75 years of patients with cancer, and that these differences might be related to the efficacy and safety of ICIs. The gut microbiota of patients aged ≥ 75 years differed from those of patients aged < 75 years. *Veillonella* was relatively more abundant in those aged < 75 years, in the SD-PR group, and in PFS ≥120 days group, indicating that *Veillonella* is an intestinal microbe, and its abundance changes with age. These changes may influence the effectiveness of ICI treatment.

One limitation of this study is that it is an observational study with limited sample size, and we have not been able to study the mechanism of the relationship between aging and ICIs efficacy by actually re-administering *Veillonella*-like gut microbiota to mice. We also did not examine potential aging-related factors such as comorbidities, medication history, or lifestyle changes including diet and exercise. Moreover, host factors potentially linked to Veillonella or Streptococcus, such as physical activity level, muscle mass, and immune parameters (e.g., T cell profiles and cytokine levels) were not assessed. Further studies with larger cohorts and more comprehensive data collection, including factors suggested by the current findings, will be necessary to clarify the relationship between gut microbiota, aging, and ICIs efficacy.

*Veillonella* is known to be abundant in marathon runners, where lactic acid produced by muscles after exercise passes through the bloodstream into the intestinal tract and produces short-chain fatty acids, including propionic acid (34). Aging may reduce *Veillonella* because muscle strength declines with age, and lactate production from muscles declines as aged individuals do not exercise; therefore, relatively less *Veillonella* is required for lactate metabolism. *Veillonella* is found not only in the intestine but also in the oral cavity. The relationship between oral and intestinal bacteria has also received recent attention (35). Reportedly, the abundance of *Veillonella* in the oral cavity is associated with a poor prognosis of small-cell lung cancer and fast tumor progression (36, 37).

*Streptococcus* was relatively more abundant in the ≥75 years group than in the <75 years group, in non-PD group in DCR and in PFS ≥ 120days group in PFS with the relative change in abundance ranking higher. *Streptococcus* was also relatively more abundant in the non-incident group of irAE than in the incident group. *Streptococcus* is an age-associated intestinal bacterium that affects the efficacy and safety of treatment. This suggests that *Streptococcus* is associated with therapeutic efficacy. *Streptococcus* increases the response of anti-Programmed cell Death 1 (PD-1)/PD- ligand 1 (L1) by inducing the wetting of CD8+ T cells in cancer tissues (38, 39). In our study, *Streptococcus* was detected more in the non-PD group, probably because *Streptococcus* enhanced the therapeutic effect of ICIs by activating cancer immunity of the host. The efficacy of ICIs may decrease because immune response usually decreases in the group of patients over 75 years of age (7–15). Therefore, in aged individuals, *Streptococcus* may activate immune cells, thereby improving the therapeutic effect of ICIs.

Although it has been reported that the diversity of the gut microbiota decreases in the elderly (29), no difference in diversity, which may affect the efficacy of cancer immunotherapy, was found between those younger than 75 years and those older than 75 years in the present study. It is possible either that the gut microbiota and its diversity differ between cancer patients and healthy individuals (40, 41) for this reason, or that the 75-year age boundary is not related to the diversity of the gut microbiota. Analysis of gene function revealed that gene expression related to aerobic respiration was higher in the Age <75 group, indicating that the gut microbiota of this group was more diverse than that of the Age ≥75 group, probably owing to the presence of relatively more aerobic bacteria in the gut microbiota. Since aerobic bacteria usually require oxygen for growth and are nondominant bacteria in the intestinal environment, oxygen content of the intestinal environment of the Age <75 group may differ from that of the Age ≥75 group.

No difference was observed in the changes of gut microbial diversity between the Age <75 and Age ≥75 groups; however, a decrease in the abundance of *Veillonella* was observed in the elderly. In addition, the analysis of DCR detected a difference in the abundance of *Streptococcus* between the non-PD and PD groups, and *Streptococcus* was relatively more abundant in the Age ≥75 group. Reports on age-related changes in the gut microbiota of patients with cancer are scanty. This study allowed us to identify changes in the gut microbiota with age in patients with cancer, and its subsequent effect on the efficacy of ICIs. In addition to assessment methods such as PS and CGA, examination of the gut microbiota, especially *Veillonella* and *Streptococcus*, may be useful as a simple and objective indicator of aging when considering the indications and limitations of anticancer therapy in the elderly.

## Data Availability

The sequence data for the human gut bacteria gene are available in the DDBJ database under accession numbers PRJDB18717.

## References

[1] Dotan E Walter LC Browner IS Clifton K Cohen HJ Extermann M. Older adult oncology, version 1.2021: Featured updates to the NCCN guidelines. JNCCN J Natl Compr Cancer Netw. (2021) 19:1006–19. doi: 10.6004/jnccn.2021.0043 34551388

[2] Chen H Cantor A Meyer J Beth Corcoran M Grendys E Cavanaugh D. Can older cancer patients tolerate chemotherapy? A prospective pilot study. Cancer. (2003) 97:1107–14. doi: 10.1002/cncr.11110 12569613

[3] Boyle HJ Alibhai S Decoster L Efstathiou E Fizazi K Mottet N. Updated recommendations of the International Society of Geriatric Oncology on prostate cancer management in older patients. Eur J Cancer. (2019) 116:116–36. doi: 10.1016/j.ejca.2019.04.031 31195356

[4] Mohile SG Dale W Somerfield MR Schonberg MA Boyd CM Burhenn PS. Practical assessment and management of vulnerabilities in older patients receiving chemotherapy: ASCO guideline for geriatric oncology. J Clin Oncol. (2018) 36:2326–47. doi: 10.1200/JCO.2018.78.8687 PMC6063790 29782209

[5] Kenis C Decoster L Van Puyvelde K De Greve J Conings G Milisen K. Performance of two geriatric screening tools in older patients with cancer. J Clin Oncol. (2014) 32:19–26. doi: 10.1200/JCO.2013.51.1345 24276775

[6] Hesketh PJ Lilenbaum RC Chansky K Dowlati A Graham P Chapman RA. Chemotherapy in patients 80 with advanced non-small cell lung cancer: combined results from SWOG 0027 and LUN 6. J Thorac Oncol. (2007) 2(6):494–8. doi: 10.1097/JTO.0b013e318060097e 17545843

[7] Ferrando-Martínez S Ruiz-Mateos E Hernández A Gutiérrez E Rodríguez-Méndez MDM Ordoñez A. Age-related deregulation of naive T cell homeostasis in elderly humans. Age (Omaha). (2011) 33:197–207. doi: 10.1007/s11357-010-9170-8 PMC3127472 20700658

[8] Nikolich-Žugich J. Ageing and life-long maintenance of T-cell subsets in the face of latent persistent infections. Nat Rev Immunol. (2008) 8:512–22. doi: 10.1038/nri2318 PMC5573867 18469829

[9] Tsukamoto H Senju S Matsumura K Swain SL Nishimura Y. IL-6-mediated environmental conditioning of defective Th1 differentiation dampens antitumour immune responses in old age. Nat Commun. (2015) 6:6702. doi: 10.1038/ncomms7702 25850032 PMC4396369

[10] Henson SM Lanna A Riddel NE Franzese O Macaulay R Griffiths SJ. P38 signaling inhibits mTORC1-independent autophagy in senescent human CD8+ T cells. J Clin Invest. (2014) 124:4004–16. doi: 10.1172/JCI75051 PMC4151208 25083993

[11] Mirza N Duque MA Dominguez AL Schrum AG Dong H Lustgarten J. B7-H1 expression on old CD8+ T cells negatively regulates the activation of immune responses in aged animals. J Immunol. (2010) 184:5466–74. doi: 10.4049/jimmunol.0903561 PMC3919800 20375308

[12] Weng NP Akbar AN Goronzy J. CD28- T cells: their role in the age-associated decline of immune function. Trends Immunol. (2009) 30:306–12. doi: 10.1016/j.it.2009.03.013 PMC2801888 19540809

[13] Lanna A Henson SM Escors D Akbar AN. The kinase p38 activated by the metabolic regulator AMPK and scaffold TAB1 drives the senescence of human T cells. Nat Immunol. (2014) 15:965–72. doi: 10.1038/ni.2981 PMC4190666 25151490

[14] Nishijima TF Muss HB Shachar SS Moschos SJ. Comparison of efficacy of immune checkpoint inhibitors (ICIs) between younger and older patients: A systematic review and meta-analysis. Cancer Treat Rev. (2016) 45:30–7. doi: 10.1016/j.ctrv.2016.02.006 26946217

[15] Landre T Taleb C Nicolas P Des Guetz G. Is there a clinical benefit of anti-PD-1 in patients older than 75 years with previously treated solid tumour? J Clin Oncol. (2016) 34:3070. doi: 10.1200/JCO.2016.34.15_suppl.3070

[16] Nosaki K Saka H Hosomi Y Baas P de Castro G Reck M. Safety and efficacy of pembrolizumab monotherapy in elderly patients with PD-L1–positive advanced non–small-cell lung cancer: Pooled analysis from the KEYNOTE-010, KEYNOTE-024, and KEYNOTE-042 studies. Lung Cancer. (2019) 135:188–95. doi: 10.1016/j.lungcan.2019.07.004 31446994

[17] Sender R Fuchs S Milo R. Are we really vastly outnumbered? Revisiting the ratio of bacterial to host cells in humans. Cell. (2016) 164:337–40. doi: 10.1016/j.cell.2016.01.013 26824647

[18] Sender R Fuchs S Milo R. Revised estimates for the number of human and bacteria cells in the body. PloS Biol. (2016) 14:e1002533. doi: 10.1371/journal.pbio.1002533 27541692 PMC4991899

[19] Caballero S Pamer EG. Microbiota-mediated inflammation and antimicrobial defense in the intestine. Annu Rev Immunol. (2015) 33:227–56. doi: 10.1146/annurev-immunol-032713-120238 PMC4540477 25581310

[20] Seki E Schnabl B. Role of innate immunity and the microbiota in liver fibrosis: crosstalk between the liver and gut. J Physiol. (2012) 590:447–58. doi: 10.1113/jphysiol.2011.219691 PMC3379693 22124143

[21] Kanazawa K Konishi F Mitsuoka T Terada A Itoh K Narushima S. Factors influencing the development of sigmoid colon cancer: Bacteriologic and biochemical studies. Cancer. (1996) 77:1701–6. doi: 10.1002/(SICI)1097-0142(19960415)77:8+<1701::AID-CNCR18>3.0.CO;2-1 8608565

[22] Spencer CN McQuade JL Gopalakrishnan V McCulloch JA Vetizou M Cogdill AP. Dietary fiber and probiotics influence the gut microbiome and melanoma immunotherapy response. Science. (2021) 374(6575):1632–40. Available online at: https://www.science.org. 10.1126/science.aaz7015 PMC8970537 34941392

[23] Routy B Le Chatelier E Derosa L Duong CPM Alou MT Daillère R. Gut microbiome influences efficacy of PD-1-based immunotherapy against epithelial tumors. Science. (2018) 359(6371):91–7. Available online at: https://www.science.org. 10.1126/science.aan3706 29097494

[24] Gopalakrishnan V Spencer CN Nezi L Reuben A Andrews MC Karpinets TV. Gut microbiome modulates response to anti-PD-1 immunotherapy in melanoma patients. Science. (2018) 359(6371):97–103. Available online at: https://www.science.org. 10.1126/science.aan4236 PMC5827966 29097493

[25] Sivan A Corrales L Hubert N Williams JB Aquino-Michaels K Earley ZM. Commensal Bifidobacterium promotes antitumor immunity and facilitates anti-PD-L1 efficacy. Science. (2015) 350:1084–9. doi: 10.1126/science.aac4255 26541606 PMC4873287

[26] Blake SJ Wolf Y Boursi B Lynn DJ. Role of the microbiota in response to and recovery from cancer therapy. Nat Rev Immunol. (2024) 24(5):308–25. doi: 10.1038/s41577-023-00951-0 37932511

[27] Hamada K Isobe J Hattori K Hosonuma M Baba Y Murayama M. Turicibacter and Acidaminococcus predict immune-related adverse events and efficacy of immune checkpoint inhibitor. Front Immunol. (2023) 14. doi: 10.3389/fimmu.2023.1164724 PMC10189048 37207204

[28] Takahashi K Nishida A Fujimoto T Fujii M. Reduced abundance of butyrate-producing bacteria species in the fecal microbial community in Crohn's disease. Digestion. (2016) 93(1):59–65. doi: 10.1159/000441768 26789999

[29] Biagi E Candela M Fairweather-Tait S Franceschi C Brigidi P. Aging of the human metaorganism: The microbial counterpart. Age (Omaha). (2012) 34:247–67. doi: 10.1007/s11357-011-9217-5 PMC3260362 21347607

[30] Mariat D Firmesse O Levenez F Guimarăes VD Sokol H Doré J. The firmicutes/bacteroidetes ratio of the human microbiota changes with age. BMC Microbiol. (2009) 9:123. doi: 10.1186/1471-2180-9-123 19508720 PMC2702274

[31] Mueller S Saunier K Hanisch C Norin E Alm L Midtvedt T. Differences in fecal microbiota in different European study populations in relation to age, gender, and country: A cross-sectional study. Appl Environ Microbiol. (2006) 72:1027–33. doi: 10.1128/AEM.72.2.1027-1033.2006 PMC1392899 16461645

[32] Biagi E Nylund L Candela M Ostan R Bucci L Pini E. Through ageing, and beyond: Gut microbiota and inflammatory status in seniors and centenarians. PloS One. (2010) 5:e10667. doi: 10.1371/annotation/df45912f-d15c-44ab-8312-e7ec0607604d 20498852 PMC2871786

[33] Odamaki T Kato K Sugahara H Hashikura N Takahashi S Xiao JZ. Age-related changes in gut microbiota composition from newborn to centenarian: A cross-sectional study. BMC Microbiol. (2016) 16:90. doi: 10.1186/s12866-016-0708-5 27220822 PMC4879732

[34] Scheiman J Luber JM Chavkin TA MacDonald T Tung A Pham LD. Meta-omics analysis of elite athletes identifies a performance-enhancing microbe that functions via lactate metabolism. Nat Med. (2019) 25:1104–9. doi: 10.1038/s41591-019-0485-4 PMC7368972 31235964

[35] Zepeda-Rivera M Minot SS Bouzek H Wu H Blanco-Miguez A Manghi P. A distinct *Fusobacterium* nucleatum clade dominates the colorectal cancer niche. Nature. (2024) 628:424–32. doi: 10.1038/s41586-024-07182-w PMC11006615 38509359

[36] Tsay JCJ Wu BG Sulaiman I Gershner K Schluger R Li Y. Lower airway dysbiosis affects lung cancer progression. Cancer Discov. (2021) 11:293–307. doi: 10.1158/2159-8290.CD-20-0263 33177060 PMC7858243

[37] Zeng W Wang Y Wang Z Yu M Liu K Zhao C. *Veillonella* parvula promotes the proliferation of lung adenocarcinoma through the nucleotide oligomerization domain 2/cellular communication network factor 4/nuclear factor kappa B pathway. Discov Oncol. (2023) 14(1):129. doi: 10.1007/s12672-023-00748-6 37452162 PMC10349017

[38] Wu H Leng X Liu Q Mao T Jiang T Liu Y. Intratumoral microbiota composition regulates chemoimmunotherapy response in esophageal squamous cell carcinoma. Cancer Res. (2023) 83:3131–44. doi: 10.1158/0008-5472.CAN-22-2593 37433041

[39] Peng Z Cheng S Kou Y Wang Z Jin R Hu H. The Gut microbiome is associated with clinical response to Anti-PD-1/PD-L1 immunotherapy in gastrointestinal cancer. Cancer Immunol Res. (2020) 8:1251–61. doi: 10.1158/2326-6066.CIR-19-1014 32855157

[40] Yang Y Misra BB Liang L Bi D Weng W Wu W. Integrated microbiome and metabolome analysis reveals a novel interplay between commensal bacteria and metabolites in colorectal cancer. Theranostics. (2019) 9(14):4101–14. doi: 10.7150/thno.35186 PMC6592169 31281534

[41] Yachida S Mizutani S Shiroma H Shiba S Nakajima T Sakamoto T. Metagenomic and metabolomic analyses reveal distinct stage-specific phenotypes of the gut microbiota in colorectal cancer. Nat Med. (2019) 25:968–76. doi: 10.1038/s41591-019-0458-7 31171880

